# Supplementary material for: Learning curve and surgical outcome of robotic assisted colorectal surgery with ERAS program
Source: Sci Rep. 2022 Nov 29;12:20566. doi: 10.1038/s41598-022-24665-w (PMC9709162; doi:10.1038/s41598-022-24665-w)
Supplement: Supplementary file 1 — Supplementary Information 1. [file 41598_2022_24665_MOESM1_ESM.zip › LSC Raw data-Ñ[▒K/063/H_ñw╝╨░O▒Kñσ.pdf]

正式摘要(電子簽章：000000134953430)

W52 / W52 ☐ 英文標題(注意:無英文姓名-請補病人基本檔!) ☐【讀取】憑證

臺中榮民總醫院

出院病歷摘要

轉入號： 轉出號：

[redacted]

(8)[redacted]	
(9)入院日期	109/01/08 (2020) 直腸外科 W52 - 143
(10)轉科日期	
(11)出院日期	109/01/30 (2020) 住院天數計 22 日

診斷

- 入院 :Rectal cancer
- 出院 :Ca of rectum, Adenocarcinoma,pT3N1a s/p Laparoscopic low anterior resection on 2020/01/16

主訴

- bowel habit change in recent months

病史

- This is a 83 year old female patient with history of
  1.Rectal cancer diagnosed 5 years ago but refuse to accept surgery, under herb
  2.Hypertension
  According to the patient, her bowel habit had change in recent months. bloody stool was also noted more frequently compare to previous. Body weight slightly decrease and feel tired very often. She came back to our hospital again and Tumor size increasing confirmed by colonoscopy. Under the impression of rectal cancer, the patient was admitted for complete survey and surgical intervention.

體檢發現

- PALAPBLE TUMOR
  - General appearance :
    - easy looking , conscious clear , without cardiopulmonary distress
    - Vital signs自動帶入date 體溫(BT) 脈搏(P) 呼吸(R) 血壓(BP) 身高(BH) 體重(BW) BMI
      20200108 36.6℃ 74次/分 19次/分 139/78mmHg 148.2cm 40.2kg 18.3
  - HEENT :
    - Eyes: no anemic conjunctiva, no icteric sclera, normal EOM
    - ENT: no otorrhea, no rhinorrhea, no erythematous mucosa, no injected tonsil, no deformity
  - Neck: supple, freely movable, trachea midline,no deformity, no engorged jugular vein , no palpable thyroid or lymph node enlargement
  - Chest:
    - Inspection: Grossly normal, symmetric and fully expansion, no accessory muscle used, no operation scar, no spider angiomata, no gynecomastia
    - Palpation: no palpable mass or nodule, no subcutaneous emphysema, no tenderness
    - Ausculation: clear breathing sound
  - Heart:
    - Percussion: normal heart size
    - Ausculation: regular heart beat, normal S1, S2, no gallop, no murmur
  - Abdomen:
    - Inspection: in
      - flat contour, symetrically, no scar, no stria, no dilated veins, no rashes
    - Ausculation: normoactive bowel sound, no abdominal bruits
    - palpation: soft, no muscle guarding or rigidity, no local tenderness, no rebounding pain, no palpable mass, no palpable pulsation
    - Percussion: no shifting dulllness, no hepatomegaly, no splenomagaly
  -

Peripheral pulsation :	CA	BA	RA	FA	PA	PTA	DPA
Right side :	++	++	++	++	++	++	++/+++
Left side :	++	++	++	++	++	++	++/+++

  - Genito-anus: no tenderness or palpable mass lesion or rectal shelf, normal external genitalia, normal anal tone, brown stool

CRS CONDITION

  -
    - Lung

手術日期及方法

- 手術日期及方法20200116 1. Laparoscopic low anterior resection, side-to-end anastomosis, EndoGIA 60/.38mm, CEEA- 29mm. Bilateral doughnut: intact, air tight test: done, no air leak.

  2. High ligation (+) above the left colic artery.
  3. Cut margin: proximal 10 cm, distal 1 cm.
  5. Take down of splenic flexure: (-).
  6. A 12mm penrose drain placed in the pelvis.

住院治療經過

- After the admission, we arranged pre op survey which showed liver metastasis. We consult GS for combine surgery but the patient refused. We arranged laparoscopic anterior resection with anastomosis on 2020/01/16. She tolerated the surgery quite well and gradually recovered. Chyle leakage was noted from drainage tube on 2020/01/20. TPN and NPO was arranged. Re try diet on 2020/01/27 and no more chyle leakage was noted. Due to her relative stable condition, the patient was discharged on 2020/01/30 and armaged OPD follow up.

合併症

- chyle leakage

一般檢查

- 累積報告 :

DATE	TIME	ALB	TP	BIL.T	BIL.D	ALKP	AST	ALT	LDH	CHOL	TG	HDL-C	LDL	UA	NH3	AMY
1090112	1817	3.7	6.1	0.3	0.1	60	21	8	209							
1090115	0654			0.3			18	9								
1090116	1711	3.4		0.4	0.1	61	25	12								
1090120	1003			0.50	0.15		19	12								
1090120	2257										354					
1090122	1150									90	71					
1090123	0914	2.5		0.26			18	5								
1090127	0935	2.8		0.26	0.10		18	5								

DATE	TIME	WBC	RBC	HGB	HCT	MCV	PLT	BLST	NEUT	BAND	SEG	LYM	MON	EOS	BAS
1090108	2319			6.2	21.1		344								
1090109	0858			9.2											
1090112	1816	7580	3.38	8.7	28.6	84.6	268		88.4			6.1	5.4	0.0	0.1
1090115	0654	2230	3.37	8.8	28.2	83.7	252		57.9			30.5	8.5	2.2	0.9
1090116	1710	8110		11.0	34.1		218		89.8			7.9	1.8	0.4	0.1
1090120	0921	6050	4.11	11.3	34.7	84.4	257		83.3			10.6	5.5	0.3	0.3
1090123	0845	4550	4.01	11.1	33.3	83.0	309		71.9			16.7	8.1	2.9	0.4
1090127	0932	8090	3.72	10.3	33.0	88.7	328		88.1			6.8	3.1	1.9	0.1

DATE	TIME	NA	K	CL	CA	BUN	CREAT	Mg	P	CRP	ESR	PCT	LIPASE	LACTATE
1090112	1800													11.1

1090112	1816													13.3
1090112	1817	133	3.5	99	9.0	24	0.86							
1090115	0652													6.5
1090115	0654	140	3.1			14	0.81							
1090116	1711	139	3.2		8.5	17	0.61							
1090120	0856													10.3
1090120	1003	133	3.4		8.5	8	0.57							
1090122	1150							1.5	2.2					
1090123	0748													7.2
1090123	0914	134	3.8	99	8.0	13	0.57							
1090127	0907													14.6
1090127	0935	133	4.7	95	8.6	12	0.59	1.7	2.9					

DATE	TIME	GLUCOSE	PAB	TRF	ZN	CU	KETONE	CK	CKMB	PT-P	PT-C	APTT-P	APTT-C	TRO-T
1090116	1712	164												

DATE	TIME	SP-GR	PH	PROT	GLUC	KETON	BILI	UROB	OB	NITR	LEU	RBC	WBC	EPCEL
1090112	1221	1.012	5.5	Negative	Negative	Negative	Negative	0.2	Negative	Negative	Trace	0-2	0-2	0-1

- 放射線報告 :檢驗項目 **:CHEST AP VIEW (PORTABLE)** 報告日期 **:1090123**

主 訴：
Result:
Finding:
> Borderline cardiomegaly
> Atherosclerotic changes of thoracic aorta.
> Prominence of right hilum
> Mildly increased insterstitial infiltration over bilateral lower lung fields.
> Left blunting CP angle, r/o pleural effusion or chronic pleural change
> Scoliosis and spondylosis of T-L spine
> On central venous catheter
Impression:
As above description.

檢驗項目 **:ABDOMEN (KUB),SUPINE** 報告日期 **:1090122**
主 訴：
Result:
Finding:
> Prominent gastric and bowel gas without definite dilatation, r/o ileus
> Obscured margin of bilateral psoas muscle and kidney shadows due to bowel gas
> Scoliosis and spondylosis of T-L spine
> Radiopaque densities at right upper abdominal region, r/o renal stones.
> Atherosclerotic changes of aorta
> clips and drainage over pelvic and lower abdomen
Impression:
As above description

檢驗項目 **:CHEST AP VIEW (PORTABLE)** 報告日期 **:1090118**
主 訴：
Result:
Finding:
> Enlarged cardiac shadow.
> Atherosclerotic changes of thoracic aorta.
> Mildly increased insterstitial infiltration over bilateral perihilar lung fields.
> Bilateral apical pleural thickening
> DJD change of thoracic and lumbar spine with osteophytes formation.
> On endotracheal tube, NG tube and EKG leads
> Subcutaneous emphysema over bilateral upper abdominal region
Impression:
As above description.

檢驗項目 **:CHEST PA VIEW** 報告日期 **:1090115**
主 訴：
Result:
Finding:
> Calcification and tortuosity of thoracic aorta, no cardiomegaly.
> Increased insterstitial infiltration over right lower lung fields
> Mild scoliosis and spondylosis of T-L spine.
Impression:
As above description.

檢驗項目 **:ABDOMEN (KUB),SUPINE** 報告日期 **:1090110**
主 訴：
Result:
Finding:
> Prominent gastric and bowel gas without definite dilatation, r/o ileus
> Obscured margin of bilateral psoas muscle and kidney shadows due to bowel gas
> Scoliosis and spondylosis of T-L spine
> Radiopaque densities at right upper abdominal region, r/o renal stones.
> Atherosclerotic changes of aorta
Impression:
As above description.

檢驗項目 **:CT ABDOMEN-SURVEY** 報告日期 **:1090113**
主 訴：
Result:
Finding:
Imaging Report Form for Colorectal Carcinoma (AJCC 8th)
1. Imaging Date: 20200109
Imaging Modality, Imaging by
CT scan
Imaging Protocol:
CT scan of whole abdomen after IV contrast medium injection was performed with oral contrast medium ingestion and reveals:
2. Tumor location / Size
Location:
Rectum
Size:
Measurable: 2.9 cm (largest diameter)
3. Tumor invasion
Tumor invades non-peritonealized pericolonic or perirectal tissues
4. Regional nodal metastasis
N1: Yes
location:
Pericolic/perirectal
5. Distant metastasis (In this study)
Single organ
6. Other findings:
> Focal asymmetrical wall thickening about 2.9cm in length at rectum with stranding of perirectal fat plane & several small perirectal lymph nodes upto 1cm in size, R/I rectal cancer. Please correlate with colonoscopy.
> Multiple ill-defined poor enhancing lesions at both lobes with largeset one about 50mm in size are noted. Liver metastases should be considered.
> Low attenuation nodules at right lobes of liver, hepatic cysts may be compatible.
> Several small low attenuation nodules at bilateral kidneys, renal cysts can not be ruled out.
> Right renal stones.

- > No obvious enlarged lymphadenopathy can be identified at paraaortic retroperitoneum.
  > No definite focal lesion in bilateral adrenal glands, pancreas and spleen
  > Atherosclerotic calcified plaque formation of thoracic and abdominal aorta and bilateral iliac arteries
  > Slightly pleural change over LLL
  > An osteosclerotic nodule at L3, R/O bone island. Suggest F/U to exclude metastasis.
  Impression:
  1. R/I rectal cancer with liver metastases. Suggest further evaluation.
  2. Other findings, as above description

特殊檢查

病理報告

其它

出院時情況

- 3.改門診治療

出院指示

藥名	劑量	劑量單位	用法	頻次	數量	數量單位	現況	天數	單位
Norvasc tab 5mg	1	TAB	PO	QD	7	TAB	TH	7	天
Gaslan tab 40mg	1	TAB	PO	TIDPRN	21	TAB	TH	7	天
Voren-K tab 25mg	1	TAB	PO	BIDPRN	14	TAB	TH	7	天
Pantoloc tab 40mg	1	TAB	PO	QDAC	7	TAB	TH	7	天

出院計劃

主治醫師 2062F 蔣鋒帆 / 住院醫師 2328K [illegible]俊凱

正式摘要(電子簽章：**000000184953430)**

W52 / W52

doctorRank:B
